# Enzymatic methods may underestimate the total serum bile acid concentration

**DOI:** 10.1371/journal.pone.0236372

**Published:** 2020-07-24

**Authors:** Kateřina Žížalová, Marek Vecka, Libor Vítek, Martin Leníček

**Affiliations:** 1 Institute of Medical Biochemistry and Laboratory Diagnostics, 1^st^ Faculty of Medicine, Charles University, Prague, Czech Republic; 2 4^th^ Department of Internal Medicine, 1^st^ Faculty of Medicine, Charles University, Prague, Czech Republic; Medizinische Fakultat der RWTH Aachen, GERMANY

## Abstract

Enzymatic assays based on bacterial 3α-hydroxysteroid dehydrogenase are the method of choice for quantification of total bile acids (BAs) in serum. Although non-specific, it is generally considered precise and robust. The aim of this study was to investigate how changes in the BA spectrum might affect the reliability of the method. We measured standard solutions of twenty-three human and murine BAs using a commercial enzymatic assay and compared the measured *vs*. expected concentrations. Additionally, total BA concentrations in rat and human cholestatic samples with an abnormal BA spectrum were measured using an enzymatic assay, and a more specific LC-MS/MS method. We observed a great variability in the response of individual BAs in the enzymatic assay. Relative signal intensities ranged from 100% in glycocholic acid (reference) to only 20% in α-muricholic acid. The enzymatic assay markedly underestimated the BA concentrations in both human and rat cholestatic sera when compared to the LC-MS/MS assay. Our study indicated that the performance of an enzymatic assay largely depends on the BA spectrum, and the total concentration of BAs can be markedly underestimated. Samples with an atypical BA spectrum (viz. in rodents) should preferably be measured by other methods.

## Introduction

For decades, serum concentrations of bile acids (BAs) have been only considered a marginal marker in clinical chemistry, reserved predominantly for laboratory diagnosis of intrahepatic cholestasis of pregnancy as well as in several rare inherited cholestatic diseases [1]. With recent advances in our understanding of their versatile metabolic, regulatory, and signaling functions (for review see [2, 3]), BA determination has become indispensable in many clinical and experimental settings.

Due to its great simplicity and availability, enzymatic determination of BAs (described by Iwata *et al*. in 1964 [4]) has represented the predominant analytical method up to the present day. It is based on bacterial 3α-hydroxysteroid dehydrogenase (3α-HSD; EC 1.1.1.50) driven oxidation of the 3α-hydroxyl group; common for virtually all BAs found in the blood serum. Substantial differences in the physicochemical properties of BAs suggest that individual BAs may vary in their reaction rates with 3α-HSD. Although an altered reaction rate may lead to a variable response, and consequently to an inaccurate quantification, only a few BAs so far have actually been tested [4, 5].

Therefore, using the standard enzymatic as well as the liquid chromatography-tandem mass spectrometry (LC-MS/MS) methods, in our current study we analyzed 23 commercially available 3α-hydroxy BAs to find out whether there were significant differences, which may affect the reliability of the enzymatic method of BA determination.

## Materials and methods

### Chemicals

Standards of cholic acid (CA), chenodeoxycholic acid (CDCA), glycocholic acid (GCA), deoxycholic acid (DCA), lithocholic acid (LCA), taurodeoxycholic acid (TDCA), glycodeoxycholic acid (GDCA), glycolithocholic acid (GLCA), glycoursodeoxycholic acid (GUDCA), hyocholic acid (HCA), taurocholic acid (TCA), and ursodeoxycholic acid (UDCA) were acquired from Sigma-Aldrich (St. Louis, MO, USA); the α-muricholic acid (α-MCA), glycochenodeoxycholic acid (GCDCA), allocholic acid (AlloCA), murideoxycholic acid (MDCA), β-muricholic acid (β-MCA), ω-muricholic acid (ω-MCA), tauro-α-muricholic acid (Tα-MCA), tauro-β-muricholic acid (Tβ-MCA), taurochenodeoxycholic acid (TCDCA), and tauroursodeoxycholic acid (TUDCA) were from Santa Cruz Biotechnology, Inc. (Dallas, TX, USA); and the hyodeoxycholic acid (HDCA) was from Supelco (Bellefonte, PA, USA). The deuterium labeled internal standards (d5-TCA, d5-GCA, d4-GCDCA, and d4-TCDCA) were purchased from Santa Cruz Biotechnology; the d4-CDCA, d4-LCA, d4-CA, d4-UDCA, d4-DCA together with ammonium acetate and formic acid (both LC-MS grade) as well as fetal bovine serum were from Sigma-Aldrich; the acetonitrile (LiChrosolv, isocratic grade) was from Merck, (Darmstadt, Germany); and the methanol (LC-MS grade) was from Biosolve BV (Valkenswaard, the Netherlands). A bile acids kit (450-A), for enzymatic determination of total BAs, was purchased from Trinity Biotech (Wicklow, Ireland).

### Standards and sera

To prepare standards for an enzymatic assay, the fetal bovine serum (serves as a matrix) was spiked with a methanolic solution of the appropriate BA in order to reach final concentrations of 20, 50, or 100 μmol/L, and then sonicated for 10 min. The methanol content was always kept below 5%. Fetal bovine serum with 5% methanol was used as the blank. Six rat cholestatic sera (randomly chosen leftovers from our previous experimental *in vivo* study [6]) and six anonymous human cholestatic sera (anonymous leftovers of cholestatic sera, that were delivered to the clinical part of our laboratory for determination of total BAs concentration) served as samples with abnormal spectra of BAs. All sera were stored at -80°C until analysis.

### Enzymatic assays of BAs

In the first reaction, 3αHSD oxidizes BAs, forming equimolar quantity of NADH. In the subsequent reaction diaphorase oxidizes NADH to NAD with concomitant reduction of nitro blue tetrazolium salt to formazan, that is quantified spectrophotometrically. Samples or standards were processed in triplicates according to the manufacturer´s instructions, and measured using an Infinite M200 plate reader (Tecan, Mannedorf, Switzerland) set to 37°C. Briefly, 50 μl of the sample was mixed with 125 μl of the test reagent and incubated for 5 min at 37°C. The reaction was terminated by adding 25 μl of Stop reagent and absorbance was read at 530 nm after 5 min incubation. Blank reaction (lacking 3α-HSD) was prepared for each sample to eliminate possible interferences. The results were either expressed as a concentration (calculated using the calibrator provided) or as a relative signal (absorbance of sample divided by absorbance of GCA at a given concentration). When needed, the incubation times were extended, as described in the Results section.

### LC-MS/MS analysis

Upon addition of the deuterated internal standards and acetonitrile deproteination of serum, BA were quantified using LC-MS/MS as previously described [7]. The total BA concentration was obtained by summing up the concentrations of all the analyzed BAs.

### Statistical analyses

Differences between total BA concentrations measured by LC-MS/MS vs enzymatically were tested using Wilcoxon signed-rank test. Reaction rates of individual BAs relative to GCA were evaluated using Mann-Whitney rank-sum test. Bonferroni correction was applied to counteract multiple testing (For clarity, p-values were corrected rather than the alpha value). Differences were considered statistically significant when the p-values were <0.05. Analyses were performed using Prism 8.0.1 software (GraphPad, San Diego, USA).

## Results

**Fig 1:**
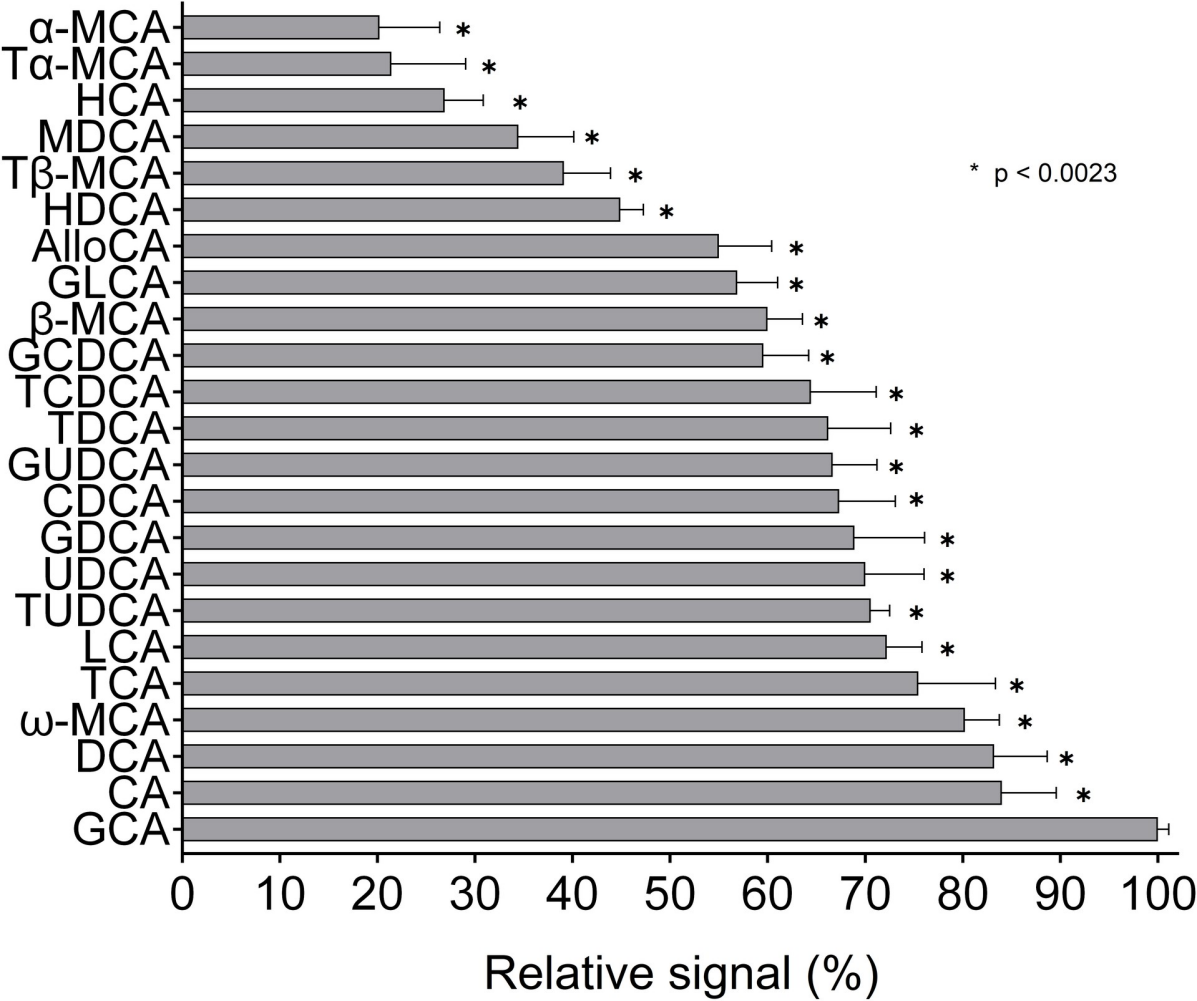
Relative signal intensities for individual BAs determined by an enzymatic method. Individual BAs were analyzed using an enzymatic kit, and the obtained signal was expressed as a % of the reference (GCA). Measurements were performed three times at three concentrations (20, 50, and 100 μmol/L)—each bar thus represents the average of nine values. All values differ significantly from reference: p <0.0023 (original p-value of <0.0001 was adjusted for 23 comparisons using Bonferroni correction).

Enzymatic determination of an individual BAs at the same concentration yielded considerably different responses. The relative signal intensities in major human BAs ranged from 100% in GCA (reference) to 60% in GCDCA. The differences were even more pronounced in minor BAs—the signal obtained from α-MCA, the “weakest” of tested BAs, reached just 20% of the reference (p<0.0023 for all comparisons, Fig 1).

**Fig 2:**
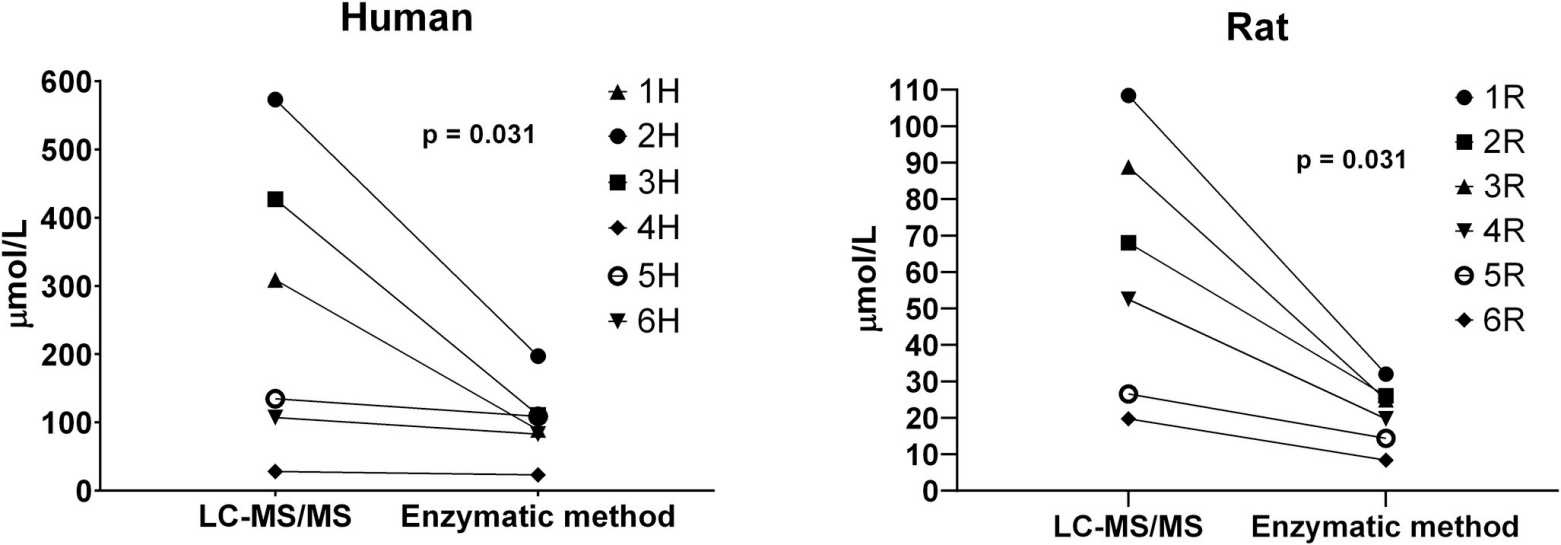
Enzymatic method underestimates total BA concentration in cholestatic serum samples with atypical BA spectra. Concentrations of total BAs in six cholestatic human sera and six cholestatic rat sera were determined using an enzymatic kit (triplicates) and LC-MS/MS (single measurement).

Such marked differences, together with the fact that the enzymatic kit uses GCA as a calibrator, prompted us to test how the total BA concentration (determined enzymatically) would differ from reality in a serum sample with an abnormal spectrum of BAs. Therefore, we analyzed six cholestatic rat and six cholestatic human sera, both enzymatically as well as using LC-MS/MS. The enzymatic kit underestimated the total BA concentration by about 45% (range 18–74%) in humans, and by 60% (range 46–72%) in rats (p = 0.031 for both groups, Fig 2).

**Fig 3:**
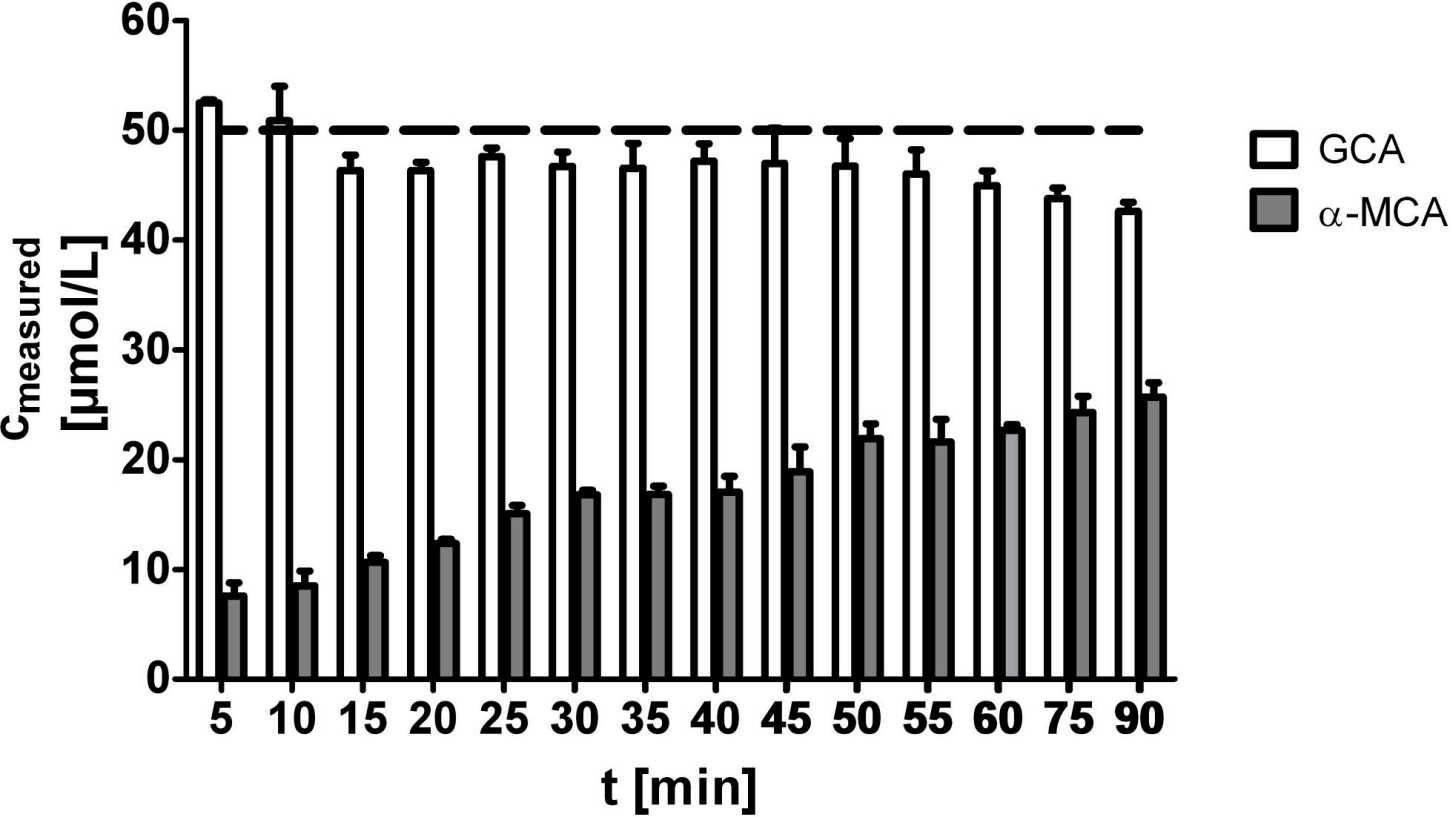
Prolonged incubation improves underestimation of poorly reacting BAs. Samples containing 50 μmol/L of either α-MCA or GCA were measured using an enzymatic kit. Incubation time varied from 5 min (recommended) up to 90 min. All measurements were done in triplicates; the dashed line represents the expected concentration.

As the reaction rates for individual BAs can differ [8], we wondered whether the performance of the enzymatic method could be improved by prolonged incubation. Therefore, we incubated α-MCA for various periods of time in order to see if the signal reached the expected value. Although the signal markedly increased (almost threefold) when incubation was prolonged to 90 min (from the 5 min that is recommended by the manufacturer), it only reached just about half of the expected value (Fig 3).

Increasing the amount of enzyme (5 times) in the reaction mixture also did not improve the performance (S1 Fig).

## Discussion

In the present study, we demonstrated the great variability of response during 3α-HSD mediated enzymatic determination of individual BAs. For major human BAs, the relative signals were within the range of 60–100%. The intensity of the signal decreased in the following order: cholic acid (CA) > deoxycholic acid (DCA) > lithocholic acid (LCA) > chenodeoxycholic acid (CDCA), which is similar to the results of previous studies [5, 8]. Free BAs tend to react faster than their glyco- or tauro-conjugated analogues, except for the most strongly reacting GCA. Importantly, we identified a group of poorly reacting BAs (mostly muricholic acids (MCAs) carrying hydroxyl group in position 6β), whose signal during enzymatic determination only reaches about 30% of what is expected. Enzymatic determination would therefore significantly underestimate the total concentration of BAs in samples rich in weakly reacting BAs (typically rodent sera). In fact, a severe underestimation was demonstrated in rat cholestatic sera, that contained about 40% of the slowest reacting BAs (MCAs, HDCA); while the fastest reacting GCA represented less than 10% of total BAs in most animals. In human cholestatic samples, the similarly severe underestimation was mostly due to abundant GCDCA or GUDCA (the later is likely present due to UDCA administration), that belong to intermediate/slow reactants (S2 Fig).

Although it has been described that prolonged incubation may completely compensate for the slower reaction rate of some BAs [8], we demonstrated that it was not sufficient for poorly reacting BAs. In the case of α-MCA, extending the incubation period from 5 to 90 min only increased the signal intensity from 20 to 50%. Further increase of the reaction time cannot be recommended, as the final reaction product is not stable, and the signal intensity decreases after about 60 min (see GCA, Fig 3).

Taken together, although enzymatic assays for measurement of total BA concentration are quite simple and straightforward, with good analytical performance [9], results depend greatly on the BA spectrum in the analyzed sample as well as on the composition of the calibrator. Commercially available calibrators typically contain strongly reacting BAs (GCA, CDCA, or TDCA) [9]. Therefore, the BA concentration in samples with an “atypical” spectrum of BAs will more-or-less be underestimated. Although such an atypical spectrum is mainly found in rodents, under pathological conditions (cholestasis [10, 11], exogenous BAs supplementation, small intestinal bacterial overgrowth [12], etc.) can be expected even in humans.

In conclusion, the performance of enzymatic assays for total BA determination in human serum seems to be appropriate for routine clinical use, where semiquantitative determination is generally sufficient. If the precise concentration is essential (mostly for research purposes), the results should be interpreted with care. In the rodent samples, enzymatic assays are far from reliable, and should be replaced by more precise analytical methods.

## Supporting information

S1 Fig Increased amount of enzyme partially improves underestimation of poorly reacting BAs. Samples containing 50 μmol/L of either α-MCA or GCA were measured using an enzymatic kit. Incubation time varied from 5 min (recommended) up to 90 min. The amount of enzyme was 5 times higher than recommended. All measurements were done in triplicates; the dashed line represents the expected concentration.
(TIF)

S2 Fig BA spectra in cholestatic samples. Serum BA spectra (measured by LC-MS/MS) of the six cholestatic rats (a) and human patients (b) are provided. BA are grouped according to their reactivity with 3α-hydroxysteroid dehydrogenase. TLCA-S (taurolithocholic acid 3-sulfate) is presented as weakly reacting BA, although it does not react at all (due to the absence of 3α-hydroxy group). BA present in ≤2% are included in “other”.
(JPG)

S1 Table Basic characteristics of LC-MS/MS method. (XLSX)
