# Supplementary figures and images for: Enzymatic methods may underestimate the total serum bile acid concentration
Source: PLoS One. 2020 Jul 24;15(7):e0236372. doi: 10.1371/journal.pone.0236372 (PMC7380613; doi:10.1371/journal.pone.0236372)

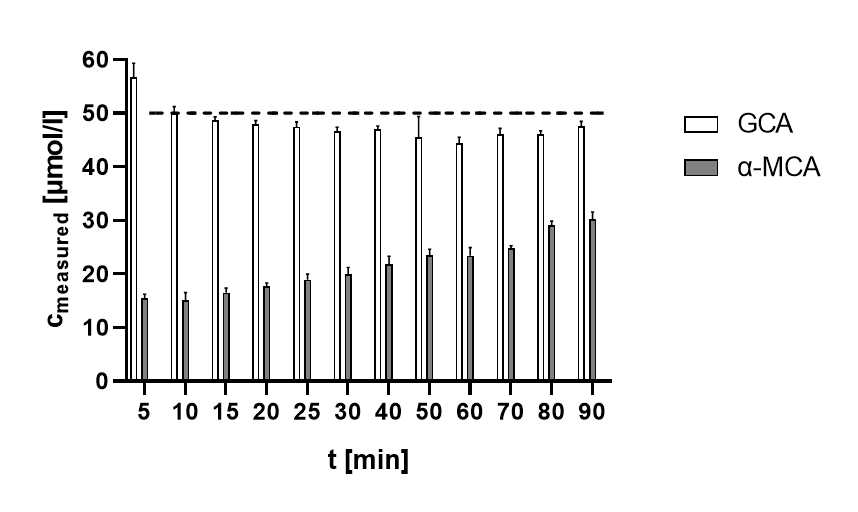

Supplement: S1 Fig — Samples containing 50 μmol/L of either α-MCA or GCA were measured using an enzymatic kit. Incubation time varied from 5 min (recommended) up to 90 min. The amount of enzyme was 5 times higher than recommended. All measurements were done in triplicates; the dashed line represents the expected concentration. (TIF) [file pone.0236372.s001.tif]

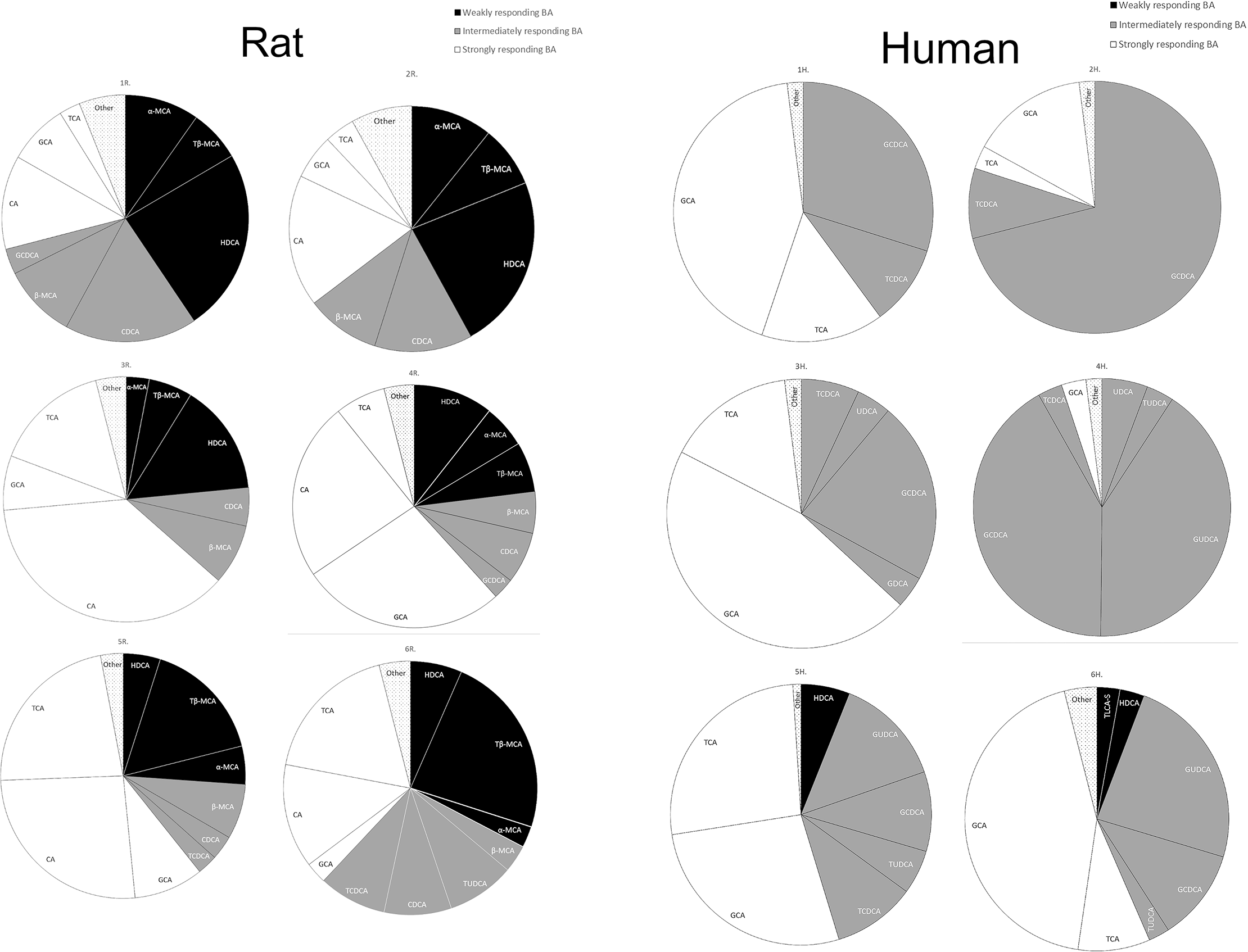

Supplement: S2 Fig — Serum BA spectra (measured by LC-MS/MS) of the six cholestatic rats (a) and human patients (b) are provided. BA are grouped according to their reactivity with 3α-hydroxysteroid dehydrogenase. TLCA-S (taurolithocholic acid 3-sulfate) is presented as weakly reacting BA, although it does not react at all (due to the absence of 3α-hydroxy group). BA present in ≤2% are included in “other”. (JPG) [file pone.0236372.s002.jpg]
